# COVID-19 and the RAAS—a potential role for angiotensin II?

**DOI:** 10.1186/s13054-020-02862-1

**Published:** 2020-04-07

**Authors:** Laurence W. Busse, Jonathan H. Chow, Michael T. McCurdy, Ashish K. Khanna

**Affiliations:** 1 Department of Medicine, Emory University, Atlanta, GA USA; 2 Emory Critical Care Center, Atlanta, GA USA; 3 Department of Critical Care Medicine, Emory Johns Creek Hospital, 6325 Hospital Parkway, Johns Creek, GA 30097 USA; 4 Department of Anesthesiology, Division of Critical Care Medicine, University of Maryland School of Medicine, Baltimore, MD USA; 5 Departments of Medicine and Emergency Medicine, University of Maryland School of Medicine, Baltimore, MD USA; 6 Department of Anesthesiology, Section on Critical Care Medicine, Wake Forest School of Medicine, Winston-Salem, NC USA; 7 Outcomes Research Consortium, Cleveland, OH USA

**Keywords:** Coronavirus, COVID-19, Angiotensin II, ACE2, RAAS

The severe acute respiratory syndrome coronavirus 2 (SARS-CoV-2) and its associated coronavirus disease 2019 (COVID-19) have wreaked havoc on healthcare systems globally. The potential for spread of this highly infectious virus, which is more transmissible and lethal than influenza, has reached pandemic proportions and has left many clinicians scrambling to provide care with scarce resources, all in the setting of no curative treatment, immunization, or effective therapy. Some candidate therapies include antivirals (remdesivir), antimalarials (hydroxychloroquine), and vaccines (mRNA-1273). Moreover, as we learn more about this virus, we have begun to draw some noteworthy conclusions regarding currently available ancillary “therapies” which may affect the natural history of the COVID-19 infection. Some of these “therapies” may actually be the avoidance of certain medications, like ibuprofen. Likewise, patients on angiotensin-converting enzyme (ACE) inhibitors or angiotensin receptor blockers (ARB) could be at a greater risk due to the mechanism by which SARS-CoV-2 enters the cell. It stands to reason that therapeutics that act counter to this mechanism may confer protection.

Angiotensin (Ang) II, the novel vasopressor agent recently approved in both the USA and Europe, may do just this. While its role as a vasopressor in shock is well known, its role in conferring protection from COVID-19, both to patients with shock and perhaps those without, is unknown and must be explored in this time of international crisis.

The level of critical illness attributable to COVID-19 has been recently described. In the recent outbreak in China, approximately 5% of patients with COVID-19 required ICU admission [1]. In Italy, the prevalence of critical illness has surpassed rates seen in China, with ICU admission required for 12% of positive cases and 16% of all hospitalized patients [2]. Critically ill patients are typically described as older with comorbidities, but cases involving young and healthy patients challenge this generalization [2, 3]. Patients are typically admitted to the ICU after 9–10 days of illness, commonly as a result of respiratory failure and acute respiratory distress syndrome (ARDS) [3]. While less common than respiratory failure, septic shock may occur in a significant portion of patients with COVID-19, and is associated with increased mortality [4]. A case series out of China described the incidence of shock in a cohort of hospitalized patients with COVID-19 to be 1.1%, but, in those with severe disease, incidence rose to 6.4% [5].

The renin-angiotensin-aldosterone system (RAAS) may be tied into the pathogenesis of the COVID-19 viral illness. The traditional RAAS pathway utilizes ACE1, primarily a pulmonary capillary endothelial enzyme, to convert AngI to AngII. As such, significant lung injury decreases the activity of pulmonary capillary endothelial-bound ACE. Initial reports from China demonstrate that approximately 40% of patients with severe illness have ARDS [5], increasing the risk for very low ACE1 function. Notably, inadequate ACE function is an independent predictor of mortality.

Specific to the SARS-CoV-2 virus, the SARS-coronavirus receptor utilizes ACE2 and the cellular protease TMPRSS2 to enter target cells (Fig. 1) [6]. The spike protein on the viral surface of SARS-CoV-2 has been shown to bind to ACE2 with 10–20 times the affinity of SARS-CoV-1, the coronavirus responsible for the SARS outbreak in 2003 [7]. The higher ACE2 affinity of SARS-CoV-2 may explain the ease of human-to-human transmission in the current pandemic [8]. Preclinical studies of novel coronaviruses (e.g., SARS-CoV-1, SARS-CoV-2) highlight that the degree of ACE2 expression directly correlates to the degree of infectivity [9, 10]. Thus, strategies to decrease ACE2 expression may attenuate the impact of SARS-CoV-2 infection.

**Fig. 1:**
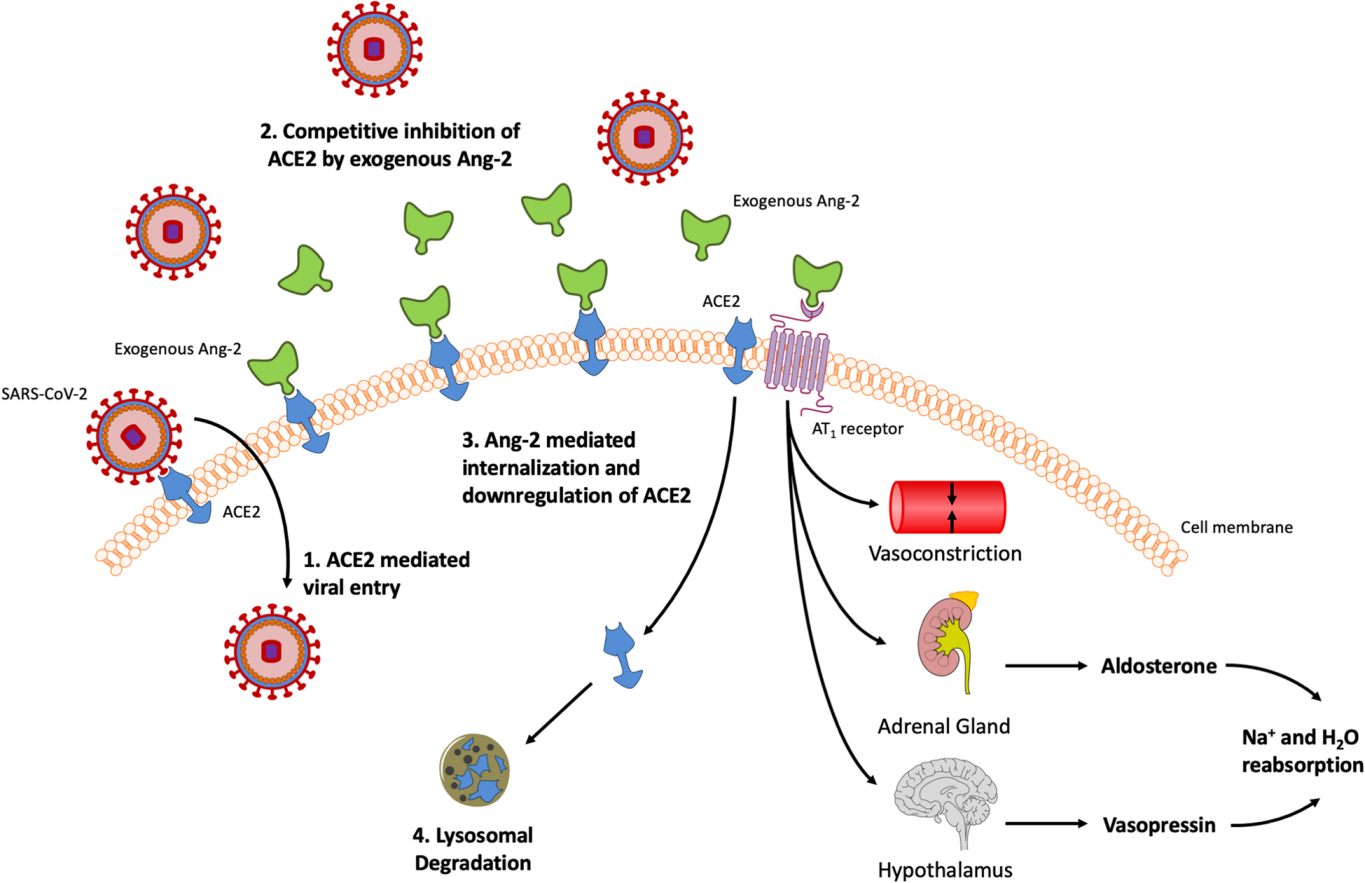
Effect of angiotensin II on the RAAS and SARS-CoV-2 binding. Angiotensin I is hydrolyzed by ACE1 to form angiotensin II, which binds to AT_1_ receptors. This causes release of aldosterone from the adrenal gland, vasopressin secretion from the hypothalamus, and vasoconstriction. Vasopressin and aldosterone both lead to increased sodium and free water reabsorption in the kidney, leading to increased mean arterial pressure (MAP). Angiotensin II is then metabolized into Ang-(1–7) by ACE2. SARS-CoV-2 binds to ACE2 to gain entry into the host cell. Exogenous angiotensin II can also bind to ACE2, which can lead to competitive inhibition of the ACE2 receptor. In addition, binding of angiotensin II to AT_1_ receptors leads to internalization, downregulation, and degradation of ACE2. These actions may potentially prevent SARS-CoV2 from entering the cell. Figure created with Motifolio Toolkit. Ang-2, angiotensin II; SARS-CoV-2, severe acute respiratory syndrome coronavirus 2; ACE1, angiotensin-converting-enzyme 1; ACE2, angiotensin-converting-enzyme 2; H_2_O, water; Na^+^, sodium

The endogenous mammalian peptide AngII is hypothesized to prevent infection from SARS-CoV-2 in multiple ways. First, because it normally binds to ACE2 during its degradation and hydrolysis into angiotensin-(1–7) [11], it may compete with the SARS-CoV-2 for the ACE2 receptor (Fig. 1). Second, the binding of AngII to the AT1 receptor has been shown to cause internalization and downregulation of ACE2 through an ERK1/2 and p38 MAP kinase pathway in both in vitro animal and in vivo human models [12, 13]. Third, AngII has been shown to cause AT1 receptor-dependent destruction of ACE2 through ubiquitination and transport into lysosomes. The competitive inhibition, downregulation, internalization, and then degradation of ACE2 may decrease the degree of viral infection by interfering with host cell entry of the virus. Much has been made of the hypothetical risk of COVID-19 in the setting ACE inhibitors and ARBs, which have been shown by multiple investigators to increase expression or activity of ACE2 [14, 15]. In fact, severe COVID-19 disease has been described in patients with conditions known to be associated with RAAS blockade therapy, such as hypertension and diabetes mellitus [5]. However, to date, the link between ACE inhibitors and ARBs and severity of illness of SARS-CoV-2 infection is purely speculative. Both the American College of Cardiology and the European Society of Cardiology have published statements advising against the discontinuation of ACE inhibitors and ARBs in SARS-CoV-2.

The support of MAP with AngII in the setting of SARS-CoV-2 infection seems physiologically rational, given the aforementioned hypotheses (Table 1). Due to the large number of critically ill SARS-CoV-2 patients, AngII has been made available in Italy, Germany and the United Kingdom for compassionate use because, despite approval by the European Medicines Agency, it is not yet commercially available in Europe. Perhaps we will learn some important lessons from these patients, so as to inform our efforts going forward. For instance, should AngII be used for all COVID-19 patients in shock? Should it be considered earlier in the course of disease, perhaps as a first-line vasopressor? Finally, and more controversially, should we evaluate the modulating effects of AngII on ACE2 for the treatment of COVID-19 in patients without shock? AngII use has been described at sub-pressor doses, and multiple studies have shown that higher levels of MAP may not be harmful. As the SARS-CoV-2 pandemic evolves, we must consider any form of therapy that may “flatten” the curve (https://www.flattenthecurve.com). The physiologic relationship between ACE2 and angiotensin II is persuasive, and given the enormity of the situation, we are obligated to explore this therapy as a potential avenue of treatment.

**Table 1:** Information supporting the use of angiotensin II in COVID-19 disease

Increased ACE2 increases infectivity of SARS [6, 10]	
Decreased ACE2 expression decreases infectivity of SARS [6, 10]	
SARS-CoV-2 utilizes ACE2 to enter cells like SARS-CoV-1 [6]	
Patients taking ACE inhibotors and ARBs have increased ACE2 expression [15]	
Exogenous angiotensin II decreases ACE2 expression [12, 13]	
Patient with hypertension are at high risk for severe COVID infection and death [5]	
Hypothesis: Exogenous angiotensin II via reduction ACE2 expression in the vasculature and heart may decrease viral propagation and thus improve outcomes.	

## Data Availability

Not applicable
